# Predicting 2,000-meter rowing ergometer performance with 7-stroke peak power, 20-Second, and 60-Second maximal effort tests in NCAA DI female rowers

**DOI:** 10.3389/fphys.2026.1791552

**Published:** 2026-07-21

**Authors:** Joseph M. DeLeo, Drake A. Eserhaut, Jessica A. Provost, Kathryn E. Ackerman, Andrew C. Fry

**Affiliations:** 1 Jayhawk Athletic Performance Laboratory – Wu Tsai Human Performance Alliance, University of Kansas, Lawrence, KS, United States; 2 Wu Tsai Female Athlete Program, Women's Health, Sports & Performance Institute, Boston, MA, United States; 3 Sport and Human Performance Research Centre, University of Limerick, Limerick, Ireland; 4 Health Sciences Program, University of Kansas, Overland Park, KS, United States; 5 Department of Neuroendocrine Massachusetts General Hospital, Boston, MA, United States; 6 Harvard Medical School, Boston, MA, United States

**Keywords:** 2kerg, anaerobic capacity, athlete monitoring, female athletes, physiological testing, rowing

## Abstract

**Purpose:**

Using methods similar to those of Cerasola et al. (2022), we sought to determine if the 20-second (20-s), and 60-second (60-s) maximal effort rowing ergometer tests serve as predictors of 2,000-meter ergometer test (2kerg) performance in a cohort of female university rowers. Secondarily, we also sought to assess if the 7-Stroke Peak Power Test (7SPP) and years of rowing experience improve the strength of prediction models.

**Methods:**

40 NCAA DI female rowers performed anaerobic capacity tests (7SPP, 20-s, 60-s) at four different time points during the 2023–2024 academic year. A 2kerg was performed three weeks after the last anaerobic capacity test date. Power (W_7SPP_, W_20_, W_60_), as well as 2kerg variables of time (T_2000_), velocity (V_2000_), power (W_2000_), and stroke length (SL_2000_), were included for analysis. An anaerobic profile of each athlete was created based on W_7SPP_, W_20_, W_60_ and normalized to W_2000_.

**Results:**

W_60_ was the best individual predictor of T_2000_ (R^2^ = 0.611, F = 59.68, p < 0.001) and W_60_ also had a very large negative correlation with T_2000_ (r = -0.78, p < 0.001). W_20_ was the second-best predictor of T_2000_ (R^2^ = 0.536, F = 43.9, p < 0.001) and it also had a very large negative correlation with T_2000_ (r = -0.73, p < 0.001). W_7SPP_ was the weakest predictor of all anaerobic capacity tests (R^2^ = 0.4171, F = 27.19, p < 0.001).

**Conclusion:**

The 60-s maximal effort rowing ergometer test is a time-efficient anaerobic capacity test that can be used to monitor rowing performance, indicate potential improvements in 2kerg performance, and confirm positive adaptations to the training program.

## Introduction

Sport scientists and coaches utilize various aspects of athlete monitoring to determine if an individual or team is positively adapting to a training program (McGuigan, 2017). This can be accomplished through many methods. Monitoring performance can be as simple as maintaining a training diary and recording athletes’ rate of perceived exertion (RPE), or as comprehensive as using athlete management software along with global positioning systems (GPS), heart rate (HR) monitors, countermovement jumps on force plates (DeLeo et al., 2025), surveys and biomarkers. However, to effectively conduct athlete monitoring, clarity is needed among coaches, athletes, and support staff (e.g., sport scientists, strength and conditioning coaches, physiologists) regarding what performance tests should be tracked, as well as the physical demands and physiological capacities required to achieve success in a given sport (Gabbett et al., 2017).

The Olympic sport of classic, or flatwater, rowing is competed at a distance of 2,000-meters (2k). Successful race performance is predicated on a combination of factors including anthropometrics (Penichet-Tomas et al., 2021; Alföldi et al., 2025), biomechanics (Warmenhoven et al., 2018; Jensen et al., 2023; Legge et al., 2024a), and various aspects of physiology (Secher, 1993; Ingham et al., 2002; van der Zwaard et al., 2018; Behm et al., 2025). The energy system contributions to a 2,000-meter ergometer test (2kerg) have been well described previously and are approximately 70 to 77% aerobic and 23 to 30% anaerobic (Hagerman, 1984; Martin and Tomescu, 2017). Therefore, it is important to test and monitor both aerobic and anaerobic capacities of rowers. Previous research has indicated that various anaerobic indices may serve as better predictors of 2k rowing performance than aerobic metrics. For example, Ingham et al. found that power at maximal oxygen consumption (W_VO2max_) (female: r = 0.91, p < 0.001; male: r = 0.93, p < 0.001; total: r = 0.95, p < 0.001) and maximal power (W_max_) (female: r = 0.74, p < 0.001; male: r = 0.88, p < 0.001; total: r = 0.95, p < 0.001) over seven strokes (two building, five maximal) were the strongest single correlates for 2kerg performance in 41 World Rowing Championship finalists (Ingham et al., 2002). This is also supported by Šmída et al., who found that a 10-Second maximal test was strongly correlated with 2,000- and 6,000-meter rowing ergometer performances (6kerg) (Šmída et al., 2017). A similarly designed study by Bourdin et al., which included 54 national and international-level French oarsmen, found that peak power [Ppeak (W), r = 0.92, p < 0.001, R^2^ = 0.846] during an incremental test had a stronger relationship to 2kerg performance than VO_2_max (r = 0.84, p < 0.001) (Bourdin et al., 2004). Legge et al. identified key physical and technical attributes for elite (n = 24) and junior (n = 28) rowers by evaluating on-water biomechanical variables, such as boat speed (meters per second, m/s) and distance per stroke (m/stroke), and on-land variables, such as the 7-Stroke Peak Power Test (7SPP) and 500-meter (500-m) average power (Legge et al., 2024b). Large to very large positive correlations were found between 7SPP and boat speed (male: r = 0.58, p < 0.01; female: r = 0.72, p < 0.01) and distance per stroke (male: r = 0.55, p < 0.05; female: r = 0.72, P < 0.01) (Legge et al., 2024b). Additionally, moderate to very large positive correlations were found between 500-m average power and boat speed (male: r = 0.62, p < 0.01; female: r = 0.74, p < 0.01) and distance per stroke (male: r = 0.49, p < 0.05; female: r = 0.75, p < 0.01) (Legge et al., 2024b). This indicates that the ergometer tests of 7SPP and 500-m average power translate well to two of the most important on-water performance outcomes: boat speed and distance per stroke, making them essential to test and monitor for both development and performance.

Anaerobic capacity tests such as the 7SPP (Ingham et al., 2002; Nugent et al., 2019; Legge et al., 2024b) as well as the 20-Second (20-s) (Cataldo et al., 2015; Cerasola et al., 2022), and 60-Second (60-s) maximal effort tests (Jensen, 2005; Jensen, 2007; Cerasola et al., 2020; Cerasola et al., 2022), are used to monitor improvements in anaerobic capacity and are highly correlated with 2kerg performance. Additionally, tests that can monitor improvements in anaerobic capacity have significant practical value for coaches and rowers. Rowing events in the Los Angeles 2028 Olympics will be contested at a distance of 1,500-meters (1.5k) (Rowbottom, 2022). Diry et al. found that when race distances shifted from 2k to 1.5k to 1,000-meters (1k), the anaerobic contribution increased for each subsequently shorter distance (Diry et al., 2022). In a recent commentary by Astridge et al., the authors predict that race times will be reduced by 90 to 120 seconds across all events with an approximately 5 to 15% increase in the relative contribution of anaerobic metabolism (Astridge et al., 2022). This means that rowing races at the LA 2028 Olympics will shift into the supramaximal domain; rowers will generate power outputs above those associated with the maximal rate of oxygen uptake (VO_2_peak) (Secher, 1993; Astridge et al., 2022). Building upon this commentary, Astridge et al. conducted a study in 33 elite rowers (10 female, 23 male) and found that a 10-stroke (5 building, 5 maximal) maximal peak power (MPP) test, defined as the average power across the 5 maximal strokes, displayed near perfect association with both 2k (r = 0.93, p < 0.001) and 1.5k ( r = 0.94, p < 0.001) erg test performance (Astridge et al., 2025). Therefore, it is important for coaches and sport scientists to effectively monitor the anaerobic capacity of developing rowers and those training for the LA 2028 Olympics to help optimize performance (Astridge et al., 2024; Astridge et al., 2025).

Physiologist and former Danish elite rower, Kurt Jensen, developed a series of aerobic and anaerobic tests across multiple times and distances to assess the physiological capacity of all three energy systems and identify capacity deficiencies to improve 2k performance (Jensen, 2005; Jensen, 2007; Rowing W, 2019). All tests were performed on the Concept2 RowErg over a 4-day period, and results were then used to create a complete power profile for the rowing athlete, providing the opportunity to assess changes in anaerobic capacity and maximal aerobic power (Jensen, 2005; Jensen, 2007; Rice, 2019a; Rowing W, 2019). Aligned with this outcome the power profile is scalable to large teams and provides coaches and sport scientists an objective way to assess the adaptation of their athletes and the effectiveness of their training program (Rice, 2019a).

Jensen’s original protocol consisted of the following tests: Day 1 – a 10-Second maximal effort test (muscle power) with the best score from three trials, followed by a 30-minute break, and a 6kerg test (aerobic capacity); Day 2 – a 2kerg test (aerobic power); Day 3 – 60-Second (60-s) maximal effort test (anaerobic capacity); Day 4 – a 60-minute test (aerobic capacity, endurance) (Jensen, 2005). Later, Jensen changed the 10-Second test to 100 meters (Jensen, 2007), which is approximately 20 seconds; this was mainly due to Concept2 software changes that prevented a time < 20-seconds or a distance < 100 meters to be programmed into the monitor. When the power output of these anaerobic tests were normalized to 2kerg power outputs (W_2000_) of elite rowers from Denmark, Italy, and the United States, performances were nearly identical (Jensen, 2007; Rowing W, 2019). For example, the 100-meter test results demonstrated a mean of 175% ± 17%, 174% ± 16%, and 188% ± 16% power relative to W_2000_ for the Danish, Italian, and USA elite male rowers, respectively (Jensen, 2007). Similarly, for the 60-s test, the mean was 154% ± 12%, 150% ± 8%, and 149% ± 3% power relative to W_2000_ for the Danish, Italian, and USA elite male rowers, respectively (Jensen, 2007). These data highlight that high levels of neuromuscular power and anaerobic capacity are necessary to compete at the elite level of rowing and performance is remarkably consistent in rowers across multiple countries.

Today, national governing bodies, (e.g., Rowing Australia) have adapted the power profile to different rowing ergometer distance (500-m, 2k, 6k, 30-minute) (Rice, 2019a). Recently, Cerasola and colleagues had 17 finalists from the Italian Men’s Junior Rowing Championship (aged 15-18 years) perform the 20-s and 60-s maximal effort tests to predict 2kerg performance (Cerasola et al., 2022). Independently, the mean power of the 20-s and 60-s tests (W_20_ and W_60_) showed a nearly perfect correlation to 2kerg mean velocity (V_2000_)_;_ W_20_ (r = 0.95, p < 0.004) and W_60_ (r = 0.98, p < 0.0005). Cerasola et al. (2022) found, when used together in stepwise multiple regression, W_20_ and W_60_ explained 96.8% of the variance (p < 0.01) in V_2000_ (Cerasola et al., 2022). However, a common thread across the work of Jensen, Ingham, Astridge, and Cerasola was the participant performance level. All of their study subjects were elite rowers of different competitive age levels (Ingham et al., 2002; Jensen, 2005; Jensen, 2007; Rowing W, 2019; Cerasola et al., 2020; Cerasola et al., 2022; Astridge et al., 2025). There is currently a gap in the literature with no clear understanding of the predictive capability of these tests in a cohort of rowers with various experience and physiological capacities.

### Purpose:

To apply methods from Cerasola et al. (2022) to a cohort of National Collegiate Athletic Association Division I (NCAA DI) female rowers and determine if the 7SPP, 20-s, and 60-s maximal effort tests serve as predictors of 2kerg performance. A secondary purpose was to determine if rowing experience improves the strength of any prediction models and to evaluate the intra-day and inter-day reliability of the selected tests.

## Methods

This study was completed during the 2023–2024 academic year at a university with an NCAA DI women’s rowing team. It is part of a multi-study project focused on long-term athlete monitoring in NCAA DI rowers.

### Participants

A convenience sample from members of a university’s women’s rowing team (WRow) were recruited for this study. A total of 40 NCAA DI female rowers (31 openweight, 9 lightweight), classified as Tier 3 (highly trained/national level) according to the McKay classification (McKay et al., 2022), participated in this study. Inclusion criteria were: female sex, age ≥ 18 years, and a current athlete on the WRow Team. Athletes not cleared by the university sports medicine staff for athletic participation were excluded. Informed consent was obtained electronically via a secure link from Qualtrics (Seattle, WA). The research protocol was approved by the University of Kansas’ Institutional Review Board (STUDY#00150421).

### Study design

The 7SPP, 20-s, and 60-s tests were part of the standard test protocols for the WRow Team and formed a longitudinal, repeated measures design. All three anaerobic capacity tests were performed at four different time points during the 2023–2024 academic year: August 29^th^, September 27^th^, November 30^th^, and January 23^rd^, with the final test date being used for analysis. The 2kerg was performed on February 13, 2024, preceding the main competitive season, beginning in March.

### 2,000-meter Erg test procedure

A standardized team warm-up was performed prior to the 2kerg test which consisted of a seven-minute dynamic warm-up on land (e.g., high knees, ankle cradle, bodyweight squat, front plank, etc.) and a 15-minute warm-up on the RowErg [4’ at 18 strokes per minute (SPM) at utilization 2 (UT2) heart rate (HR), 4’ at 20 SPM at UT2 HR, 1’ at utilization 3 (UT3) HR, 1’ at Target Goal Pace, 1’ at UT3 HR, 1’ at 2-3 splits faster than target goal pace, 1’ at UT3 HR, 1’ at Target Goal Pace, and 1’ at UT3 HR]. Testing was done in coordination and cooperation with the WRow coaching staff to ensure it aligned with the training program and their periodization structure. As such the training intensity distribution (TID) followed a polarized model with ~ 70-80% below the first lactate threshold (LT_1_) and ~ 20-30% above the second lactate threshold (LT_2_) (Seiler and Tønnessen, 2009; Stöggl and Sperlich, 2015; Stöggl, 2018). The total training volume for the weeks of Jan 22^nd^ – 28^th^, Jan 29^th^ – Feb 4^th^, and Feb 5^th^ – 11^th^ was 436, 546, and 472 minutes which included at least one threshold session and one high-intensity interval training session. Each week the team completed 2 x 45 minutes for strength and conditioning sessions.

### Anaerobic capacity tests procedure

The anaerobic capacity tests were performed in the following order: 7SPP, 20-s test, and the 60-s maximal effort test. Prior to the tests, athletes performed a dynamic warm-up followed by a 10-minute rowing ergometer warm-up based on USRowing’s recommended guidelines (USRowing, 2022). In alignment with team protocol, the drag factor was set to 110 for openweight athletes and 100 for lightweight athletes. The use of different drag factors for sex and weight class is a commonly accepted training and testing practice within the sport of rowing (Rice, 2019a; Rice, 2019b; Rice, 2019c). A prior investigation looked at peak power output at three different drag factors (Metikos et al., 2015) in physically inactive participants, physically active participants, and rowers. The rowers relative peak power output (W/kg) was incredibly consistent at all three drag factors: 90 drag factor (23.5 ± 5.2 W/kg), 125 drag factor (23.8 ± 5.0 W/kg), and 200 drag factor (23.3 ± 5.1 W/kg) indicating that in trained athletes they can achieve similar outputs at various drag factors (Metikos et al., 2015). The athletes in our cohort used the same drag factor for all tests across the year. All testing was performed on a Concept2 RowErg (Model D; Concept2, Inc. Morrisville, VT, USA). All tests were loaded onto the Concept2 PM5 Monitor via Ludum (Cambridge, UK). Verbal encouragement was provided by fellow athletes and coaches throughout testing.

### 7-stroke peak power test

Athletes completed three 7SPP trials separated by 4 minutes of passive rest between each trial to ensure the fly wheel came to a dead stop (Nugent et al., 2019); with no cap for stroke rate. The highest power, (W – watts) value on a single stroke during the 7SPP trial was recorded as the athletes’ peak power (W_7SPP_). This single stroke value was manually recorded by a coach or coxswain and recorded into a spreadsheet. For the 7SPP test, the PM5 monitor was set to 20-s.

### 20-Second and 60-Second maximal effort tests

Rowers were instructed to achieve the highest mean power output (W_20_ and W_60_) across the 20-s and 60-s tests. Five minutes of passive rest separated the 20-s and 60-s tests. There was no stroke rating cap for the 20-s test and after the first 5 strokes in the 60-s test rowers had to settle to a rate ≤ 40 strokes per minute, per USRowing guidelines (USRowing, 2022).

Note of clarity to reader: When referring to the tests we use 7SPP, 20-s, and 60-s. When referring to the respective power for each test we use W_7SPP_, W_20_, and W_60_.

### Statistical analyses

The test date closest to their 2kerg was used in the analysis to mitigate any differences in fitness compared to test dates conducted earlier in the year. All rowing ergometer data were downloaded as Microsoft Excel files from Ludum’s cloud-based rowing athlete management software system.

Statistical analyses and data visualizations were performed in RStudio (2025.05.0 + 496) (team P, 2025) using the following key analytical packages: tidyverse (v. 2.0.0) (Wickham et al., 2019), car (v. 3.1-3) (Fox and Weisberg, 2019), psych (v. 2.5.6) (Revelle, 2025), gtsummary (v. 2.4.0) (Sjoberg et al., 2021), gt (v. 1.1.0) (Iannone et al., 2025), and corrplot (v. 0.95) (Simko and WaV, 2024).

**Figure 1:**
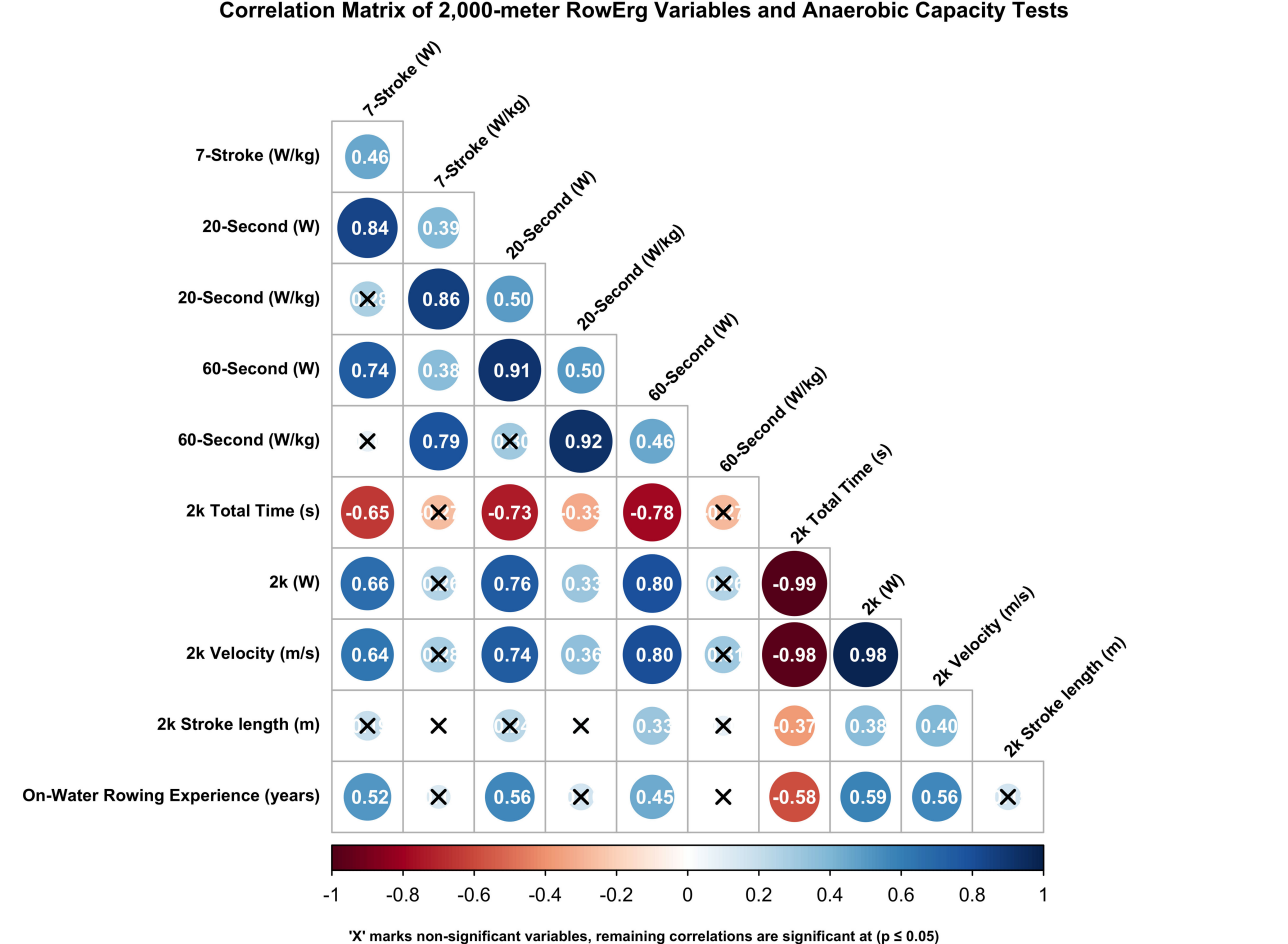
Heatmap correlation matrix.

Statistical significance was set *a priori* to α = 0.05. Variables of interest were checked for normality using the Shapiro-Wilk test and visually using Normal Q-Q Plots. Continuous variables were evaluated descriptively using means and standard deviations (x̅ ± SD) for normally distributed data and median [interquartile range (Q1, Q3)] for non-normally distributed data. Pearson product-moment coefficient of correlation (r) determined the association between W_7PP_, W_20_, W_60_, W_2000_, V_2000_, T_2000_, and SL_2000_ and their relative values. The strength of the Pearson correlation and coefficient of determination (R^2^) values were evaluated using Hopkins’ scale of magnitudes (Hopkins, 2002); trivial (r = 0.0, R^2^ = 0.0 – 0.01), small (r = 0.1 – 0.3, R^2^ = >0.01 – 0.09), moderate (r = 0.3 – 0.5, R^2^ = >0.09 – 0.25), large (r = 0.5 – 0.7, R^2^ = >0.25 – 0.49), very large (r = 0.7 – 0.9, R^2^ = >0.49 – 0.81), nearly perfect (r = 0.9, R^2^ = >0.81) and perfect (r = 1, R^2^ = 1.0); please see **Figure 1** for the heatmap correlation matrix.

**Figure 2:**
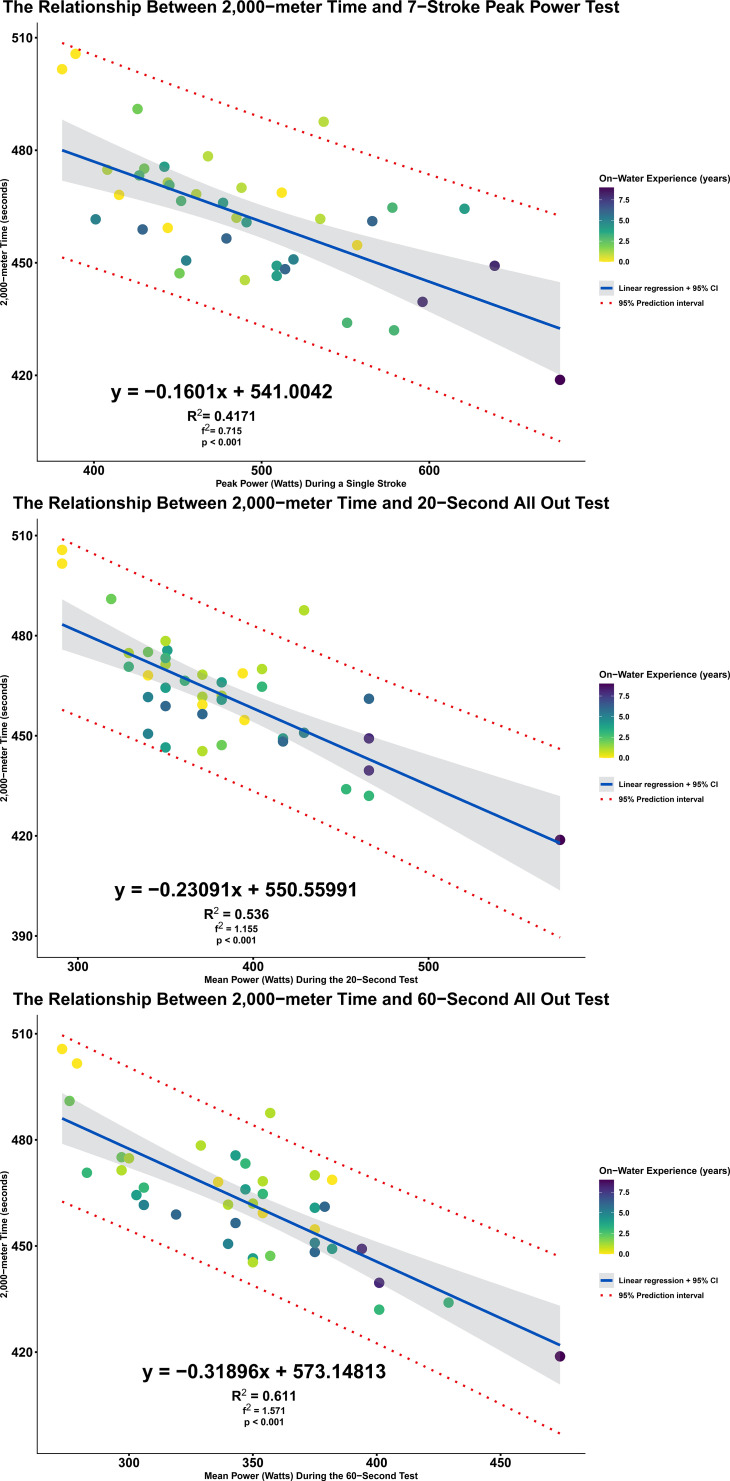
Regression model plots for 7spp, 20-Second, 60-Second.

Linear regression was performed for each anaerobic capacity test (i.e., 7SPP, 20-s, 60-s) independently as well as replicating the stepwise multiple-regression model proposed by Cerasola and colleagues (Cerasola et al., 2022). Furthermore, a new stepwise multiple-regression model that included all three anaerobic capacity tests was analyzed to determine if this could strengthen the predictive model. Residuals vs. Fitted, Scale-Location, and Cook’s Distance were used to test model fit by testing the constant variance assumption, potential outlier influence, and whether the linear relationship was valid. Lastly, we evaluated the potential impact of rowing experience on these anaerobic capacity tests by adding this into the stepwise multiple-regression models and using color to distinguish each rowers’ experience as shown in **Figure 2**.

Sample size was fixed by the roster of the team and the athletes who were available to complete testing (N = 40). Therefore, a sensitivity analysis was conducted *post-hoc* using G*Power 3.1 (v. 3.1.9.6) instead of an *a priori* power analysis (Faul et al., 2009; Lakens, 2022). A sensitivity power analysis allows sample size and error rates to be fixed to evaluate power across a range of effect sizes (Mesquida et al., 2023). Given these constraints we used conventional (power = 0.80) and conservative (power = 0.95) assumptions with an α = 0.05 and number of predictors set to four. Cohen states the following thresholds for f^2^: 0.02, 0.15, and 0.35 which represent small, medium, and large local effect sizes, respectively (Cohen, 1988). The calculated local effect sizes from G*Power based on our criteria were f^2^ = .34 (power = 0.80) and f^2^ = .53 (power = 0.95) both large local effect sizes. Next, the effect sizes (f^2^) of each regression model were calculated from the equation f^2^ = R^2^/1 - R^2^ (Cohen, 1988; Selya et al., 2012) to determine if they were greater or less than the conventional (f^2^ = .34, power = 0.80) or conservative (f^2^ = .53, power = 0.95) sensitivity analysis values. Finally, variance inflation factor (VIF) was provided for all multiple regression models to account for potential multicollinearity of any variables that may have a high inter-correlation (James et al., 2013). VIF Thresholds were as follows: 1 = no collinearity; >1 VIF < 5 = moderately correlated; >5 VIF < 10 = high correlation that may be problematic; and VIF > 10 regression coefficients are poorly estimated due to multicollinearity (Akinwande et al., 2015).

The anaerobic capacity tests of 7SPP, 20-s, and 60-s had reliability assessed for systematic bias, within-individual variation, test-retest correlation, and an estimate of the smallest measurement error (Hopkins, 2000; Riemann and Lininger, 2018). First, test-retest reliability was assessed using Intraclass Correlation Coefficients (ICC^3,1^) modeled as a two-way mixed-effects model and using restricted maximum likelihood (REML) estimation to account for any athletes who missed test dates (Shrout and Fleiss, 1979) and because we were evaluating > 2 trials (Hopkins, 2000). ICC^3,1^ thresholds were as follows: <0.5 poor reliability; 0.5-0.75 = moderate reliability; 0.75-0.9 = good reliability; and > 0.9 = excellent reliability (Koo and Li, 2016). Typical Error (TE) was calculated (SD_diff_ / √2, averaged across consecutive pairs) to determine the random variability of a single individual’s values on repeat testing (Hopkins, 2000) 95% Confidence Intervals (95%CI) calculated from chi-squared distribution (Tate and Klett, 1959). Minimal Detectable Change at 95% confidence (MDC_95_) was calculated (TE x 1.96 x √2)to establish boundaries for TE which delineates a clear change when values are outside this range (Riemann and Lininger, 2018). Lastly, Coefficient of Variation (CV%) was calculated to provide a dimensionless measure to compare across test dates, the three anaerobic capacity tests, and individual athletes. This was done using two methods to allow direct comparison to Astridge (Astridge et al., 2025) and Nugent (Nugent et al., 2019). First, we used the equation (SD_diff/_mean) x 100; to allow comparison across all four sessions (**Table 1**) and pairwise comparison between sessions (**Table 2**) (Astridge et al., 2025). The second CV equation was (SD/mean) x 100; where the standard deviation and mean represented each individual athlete’s values for their 7-stroke trial. Finally, absolute W_7PP_, W_20_, and W_60_ were expressed as a percentage of W_2000_ when comparing the anaerobic profile of each rower.

**Table 1:** Test-retest reliability of anaerobic capacity tests.

Anaerobic Tests	ICC(3,1)*^1^*	TE (W)*^2^*	MDC_95_ (W)*^3^*	CV (%)*^4^*
7-Stroke Peak Power	0.854 [0.778, 0.912]	27.8 [23.6, 33.9]	77.1 [65.4, 93.9]	8.01 [6.80, 9.75]
20-Second	0.825 [0.738, 0.894]	23.4 [19.9, 28.5]	64.9 [55.1, 79.0]	8.63 [7.33, 10.51]
60-Second	0.776 [0.671, 0.861]	19.6 [16.6, 23.9]	54.4 [46.1, 66.2]	7.91 [6.71, 9.63]

*^1^*ICC (3, 1) = (MSB − MSE) / (MSB + (*k* − 1) MSE), where MSB = between-subjects mean square, MSE = error mean square, *k* = number of sessions; two-way mixed-effects model, consistency definition (Shrout and Fleiss, 1979); REML estimation used to accommodate missing sessions.

*^2^*TE = SDdiff / √2, averaged across consecutive session pairs (Hopkins, 2000); 95% CI via chi-squared method (Tate and Klett, 1959).

*^3^*MDC_95_ = TE × 1.96 × √2 (Riemann and Lininger, 2018).

*^4^*CV(%) = (SDdiff / Mean) × 100, where SDdiff is the standard deviation of paired difference scores between sessions, and Mean = grand mean of all scores across sessions (Astridge et al., 2025).

ICC, Intraclass Correlation Coefficient; CI, Confidence Interval; TE, Typical Error; MDC_95_, Minimal Detectable Change at 95% confidence; CV, Coefficient of Variation; W, Watts. Reliability Statistics Across All Four Test Dates; Values Presented as Point Estimate [95% CI].

**Table 2:** Pairwise between-session reliability by test date combination.

Anaerobic Tests	N	ICC (3, 1)^1^	TE (W)^2^	MDCss (W)^3^	CV (%)^4^
7-Stroke Peak Power					
Date 1 vs. Date 2	13	0.790 [0.445, 0.931]	29.8 [21.3, 49.1]	82.5 [59.1, 136.2]	8.38 [6.01, 13.83]
Date 1 vs. Date 3	24	0.903 [0.789, 0.957]	20.4 [15.8, 28.6]	56.5 [43.9, 79.2]	5.82 [4.52, 8.16]
Date 1 vs. Date 4	29	0.881 [0.762, 0.942]	23.2 [18.4, 31.4]	64.3 [51.0, 87.0]	6.61 [5.25, 8.94]
Date 2 vs. Date 3	18	0.752 [0.451, 0.899]	37.5 [28.1, 56.2]	103.9 [77.9, 155.7]	10.91 [8.19, 16.35]
Date 2 vs. Date 4	24	0.763 [0.527, 0.890]	37.8 [29.4, 53.0]	104.8 [81.4, 147.0]	11.03 [8.57, 15.48]
Date 3 vs. Date 4	32	0.946 [0.892, 0.973]	16.2 [13.0, 21.6]	44.9 [36.0, 59.7]	4.63 [3.71, 6.16]
20-Second					
Date 1 vs. Date 2	13	0.792 [0.449, 0.932]	22.5 [16.1, 37.1]	62.3 [44.7, 102.9]	8.24 [5.91, 13.60]
Date 1 vs. Date 3	23	0.874 [0.726, 0.944]	19.1 [14.8, 27.1]	53.0 [41.0, 75.0]	6.88 [5.32, 9.74]
Date 1 vs. Date 4	28	0.852 [0.706, 0.929]	21.1 [ 16.7, 28.7]	58.4 [46.2, 79.5]	7.57 [5.99, 10.31]
Date 2 vs. Date 3	18	0.830 [0.601, 0.933]	23.1 [17.4, 34.7]	64.1 [48.1, 96.1]	8.78 [6.59, 13.17]
Date 2 vs. Date 4	24	0.717 [0.449, 0.867]	29.4 [22.9, 41.3]	81.6 [63.4, 114.5]	11.31 [8.79, 15.87]
Date 3 vs. Date 4	32	0.811 [0.647, 0.903]	24.7 [19.8, 32.8]	68.3 [54.8, 90.9]	9.01 [7.23, 11.98]
60-Second					
Date 1 vs. Date 2	13	0.740 [0.343, 0.913]	17.2 [12.3, 28.4]	47.7 [34.2, 78.7]	7.12 [5.11, 11.76]
Date 1 vs. Date 3	23	0.880 [0.738, 0.947]	14.2 [11.0, 20.1]	39.3 [30.4, 55.6]	5.56 [4.30, 7.88]
Date 1 vs. Date 4	28	0.845 [0.693, 0.925]	16.6 [13.1, 22.6]	46.0 [36.4, 62.6]	6.58 [5.20, 8.96]
Date 2 vs. Date 3	18	0.621 [0.230, 0.839]	23.9 [17.9, 35.8]	66.2 [49.7, 99.3]	9.86 [7.40, 14.78]
Date 2 vs. Date 4	24	0.592 [0.256, 0.800]	26.3 [20.4, 36.9]	72.8 [56.6, 102.2]	11.11 [8.63, 15.58]
Date 3 vs. Date 4	32	0.809 [0.644, 0.902]	17.7 [14.2, 23.6]	49.1 [39.4, 65.3]	7.06 [5.66, 9.38]

^1^ ICC(3,1) computed on each date pair via listwise deletion; *n* varies by pair.

^2^ TE= SD_diff_ / √2 (Hopkins, 2000); 95% Cl via chi-squared method (Tate and Klett, 1959).

^3^ MDC9s = TE x 1.96 x / √2 (Riemann and Lininger, 2018).

^4^ CV(%)= (SD_diff_ / Mean) x 100, where Mean= mean of both sessions in each pair (Astridge et al., 2025).

*n*, sample size with complete data for both dates; ICC, Intraclass Correlation Coefficient; TE, Typical Error; MDC9s, Minimal Detectable Change at 95% confidence; CV, Coefficient of Variation; W, Watts. Date 1, 08/29/23; Date 2, 09/27/23; Date 3, 11/30/23; Date 4, 01/23/24 - Values as Point Estimate [95% Cl].

## Results

Descriptive statistics for the participant and their test performances are provided in **Table 3**.

**Table 3:** Participant characteristics (N = 40).

Variable	Mean ± SD	Shapiro–Wilk p	Range
**Age (years)*^1^***	20.0 (18.5, 20.0)	p=0.001	18.0 – 22.0
**Height (cm)**	176.2 (± 5.4)	p=0.248	165.1 – 188.0
**Weight (kg)**	79.1 (± 11.3)	p=0.217	61.1 – 107.4
**On-Water Rowing Experience (Years)*^1^***	3.0 (1.0, 4.5)	p=0.008	0.0 – 9.0
**Indoor Rowing Experience (Years)*^1^***	3.0 (1.0, 5.0)	p=0.015	0.0 – 9.0
**7-Stroke Peak Power Test (W)**	492.0 (± 70.6)	p=0.134	381.0 – 678.0
**7-Stroke Peak Power Test (W/kg)**	6.3 (± 0.9)	p=0.930	4.2 – 8.5
**20-Second All Out Test (W)*^1^***	371.0 (350.0, 411.0)	p=0.005	291.0 – 575.0
**20-Second All Out Test (W/kg)**	4.9 (± 0.7)	p=0.479	3.2 – 6.2
**60-Second All Out Test (W)**	347.7 (± 42.9)	p=0.184	273.0 – 474.0
**60-Second All Out Test (W/kg)**	4.5 (± 0.7)	p=0.107	3.1 – 5.6
**2,000-meter (W)**	228.9 (± 26.2)	p=0.418	173.0 – 305.0
**2,000-meter (W/kg)**	2.9 (± 0.4)	p=0.984	2.0 – 3.8
**2,000-meter Speed (m/s)**	4.3 (± 0.2)	p=0.086	4.0 – 4.8
**2,000-meter Stroke Length (m)**	1.2 (± 0.1)	p=0.915	1.1 – 1.4
**2,000-meter Time (s)**	462.3 (± 17.5)	p=0.608	418.8 – 505.7

*^1^*Denotes non-normally distributed variable, reported as Median (Q1, Q3); Shapiro-Wilk p ≤ 0.05.

Abbreviations: cm = centimeters, kg = kilograms, W = Watts, W/kg = Watts per Kilogram, m/s = meters per second, m = meters, s = seconds

Findings revealed that W_60_ had a very large negative correlation (r = -0.78, p < 0.001) and was the best individual predictor for T_2000_ (R^2^ = 0.611, F = 59.7, p < 0.001, f^2^ = 1.5707); T_2000_ = 573.14813 – (0.31896 * W_60_) (p < 0.001). W_20_ also had a very large negative correlation (r = -0.73, p < 0.001) and was the second-best predictor of T_2000_ (R^2^ = 0.536, F = 43.9, p < 0.001, f^2^ = 1.1554). W_7SPP_ had the lowest correlation, albeit a large negative one, (r = -0.65, p < 0.001) and was the weakest predictor of all anaerobic capacity tests (R^2^ = 0.417, F = 27.2, p < 0.001, f^2^ = 0.7154). **Figure 1** provides a heatmap correlation matrix of 2kerg variables and all of the anaerobic capacity tests. All three of these models showed local effect sizes greater than our conservative estimate (f^2^ = 0.53) with W_60_ and W_20_ showing effect sizes that were more than double this value indicating a large effect for these two tests (Cohen, 1988) but importantly, only W_60_ showed significance in every multiple regression model. **Figure 2** show the linear regression models for the respective power of each anaerobic capacity test and T_2000_.

Using the Cerasola stepwise multiple regression model to predict T_2000_ did not significantly improve the variance of the model (R^2^ = 0.613, Adj. R^2^ = 0.592, F = 29.3, p < 0.001, f^2^ = 1.5841, VIF = 6.02). Furthermore, when switching the dependent variable from T_2000_ in the Cerasola model to V_2000_, the variance explained increased slightly, improving the model (R^2^ = 0.642, Adj. R^2^ = 0.622, F = 33.1, p < 0.001, f^2^ = 1.7898, VIF = 6.02). Additionally, including all 3 tests (i.e., 7SPP, 20-s, 60-s) in a stepwise model did not substantially improve the model’s ability to predict T_2000_ (R^2^ = 0.621, Adj. R^2^ = 0.589, F = 19.6, p < 0.001, f^2^ = 1.6364, VIF = 9.48) or V_2000_ (R^2^ = 0.646, Adj. R^2^ = 0.616, F = 21.9, p < 0.001; f^2^ = 1.8244, VIF = 9.48). All multiple regression models also showed large effect sizes that were more than double the conservative estimate (f^2^ = 0.53). However, the VIF values for two multiple regression models were > 5 indicating that multicollinearity is present and somewhat problematic. The two models including all three anaerobic capacity tests had VIF values approaching 10, indicating that these regression coefficients are poorly estimated due to multicollinearity, thus making it more difficult to isolate the contributions of W_7SPP_, W_20_, and W_60_. **Table 4** shows a summary and comparison of all the intercepts and coefficients for each linear regression model.

**Table 4:** Summary and comparison of all linear regression models.

Characteristic	7SPP			20-s			60-s			20-s + 60-s 2k time				20-s + 60-s 2k velocity				7SPP + 20-s + 60-s 2k time				7SPP + 20-s + 60-s 2k velocity			
	Beta	95% Cl	p-value*^1^*	Beta	95% Cl	p-value*^1^*	Beta	95% Cl	p-value*^1^*	Beta	95% Cl	p-value*^1^*	VIF	Beta	95% Cl	p-value*^1^*	VIF	Beta	95% Cl	p-value*^1^*	VIF	Beta	95% Cl	p-value*^1^*	VIF
(Intercept)	541.0042	510.1234, 571.8849	**<0.001**	550.5599	523.3052, 577.8146	**<0.001**	573.1481	543.8752, 602.4210	**<0.001**	572.1604	542.1987, 602.1221	**<0.001**		3.2744	3.0039, 3.5448	**<0.001**		576.3312	544.6409, 608.0215	**<0.001**		3.2448	2.9576, 3.5319	**<0.001**	
W_7SPP_	-0.1601	-0.2222, -0.0979	**<0.001**															-0.0407	-0.1375, 0.0561	0.4	3.52	0.0003	-0.0006, 0.0012	0.5	3.52
W_20_				-0.2309	-0.3015, -0.1604	**<0.001**				-0.0348	-0.1952, 0.1255	0.7	6.02	0.0002	-0.0013, 0.0016	0.8	6.02	0.0166	-0.1856, 0.2188	0.9	9.48	-0.0002	-0.0020, 0.0016	0.8	9.48
W_60_							-0.3190	-0.4025, -0.2354	**<0.001**	-0.2778	-0.4853, -0.0703	**0.010**	6.02	0.0029	0.0010, 0.0047	**0.004**	6.02	-0.2887	-0.4988, -0.0787	**0.008**	6.11	0.0029	0.0010, 0.0048	**0.003**	6.11
R^2^	0.417			0.536			0.611			0.613				0.642				0.621				0.646			
Adjusted R2	0.402			0.524			0.601			0.592				0.622				0.589				0.616			
Statistic	27.2			43.9			59.7			29.3				33.1				19.6				21.9			
Cohen's f^2^	0.7154			1.1554			1.5707			1.5841				1.7898				1.6364				1.8244			

^1^ Significance set to p ≤ 0.05 values in bold denote significance.

95%CI, 95% Confidence Interval; VIF, Variance Inflation Factor; W, Watts; s, seconds; R2, Coefficient of Determination; Statistic, F-Statistic; Cohen's f2, Effect Size of Regression Model; 7SPP, 7-Stroke Peak Power; W_7spp_, 7-Stroke Peak Power output (W); W_20_, 20-Second mean power output (W); W_60_, 60-Second mean power output (W).

Rowing experience had a notable impact on each model that included T_2000_ as the dependent variable, as every model’s variance improved when including on-water rowing experience into the stepwise multiple regression. For example, the model that includes only W_7SPP_ had R^2^ and Adj. R^2^ improving from 0.417 to 0.500 and from 0.402 to 0.473 (p < 0.001, VIF = 1.37, f^2^ = 1.0011), respectively. When expanding to the Cerasola model, the pattern continued with R^2^ and Adj. R^2^ improving from 0.613 to 0.683 and from 0.592 to 0.657 (p < 0.001, VIF = 7.37, f^2^ = 2.1542). Finally, the full model was nearly identical with R^2^ and Adj. R^2^ improving to 0.688 and 0.652 (p < 0.001, VIF = 10.55, f^2^ = 2.2028). This demonstrates that not only the quantity (number of years), but the quality of experience (competitive level) could be a factor in the relationship between these anaerobic capacity tests and 2kerg performance. This is supported by Cerasola, Ingham, Astridge, and Jensen, as all their cohorts were comprised of national team level rowers (Ingham et al., 2002; Jensen, 2005; Jensen, 2007; Cerasola et al., 2020; Cerasola et al., 2022; Astridge et al., 2025). Finally, the introduction of rowing experience had a notable reduction in VIF values and an increase in effect sizes, highlighting the impact this variable has on the strength of the models and their predictive capability. **Table 5** provides a synopsis of all regression models with rowing experience.

**Table 5:** Summary and comparison of rowing experience models (2k total time).

	7SPP + exp				20-s + exp				60-s + exp				20-s + 60-s + exp				7SPP + 20-s + 60-s + exp			
Characteristic	Beta	95% CI	p-value*^1^*	VIF	Beta	95% CI	p-value*^1^*	VIF	Beta	95% CI	p-value*^1^*	VIF	Beta	95% CI	p-value*^1^*	VIF	Beta	95% CI	p-value*^1^*	VIF
(Intercept)	527.0456	495.8872, 558.2040	**<0.001**		539.1383	510.0722, 568.2044	**<0.001**		561.0576	532.6196, 589.4956	**<0.001**		561.2123	532.5874, 589.8371	**<0.001**		564.7239	534.2966, 595.1512	**<0.001**	
W_7SPP_	-0.1169	-0.1851, -0.0487	**0.001**	1.37													-0.0323	-0.1217, 0.0570	0.5	3.54
Rowing Exp (years)	-2.4138	-4.3840, -0.4437	**0.018**	1.37	-1.7613	-3.6403, 0.1177	0.065	1.47	-2.0687	-3.5830, -0.5544	**0.009**	1.25	-2.3427	-4.0286, -0.6568	**0.008**	1.53	-2.2999	-4.0025, -0.5972	**0.010**	1.54
W_20_					-0.1871	-0.2699, -0.1044	**<0.001**	1.47					0.0619	-0.1010, 0.2249	0.4	7.37	0.1010	-0.0954, 0.2974	0.3	10.55
W_60_									-0.2662	-0.3525, -0.1799	**<0.001**	1.25	-0.3324	-0.5269, -0.1378	**0.001**	6.28	-0.3400	-0.5372, -0.1429	**0.001**	6.35
R²	0.500				0.577				0.678				0.683				0.688			
Adjusted R²	0.473				0.554				0.660				0.657				0.652			
Statistic	18.5				25.3				38.9				25.9				19.3			
Cohen's f²	1.0011				1.3655				2.1030				2.1542				2.2028			

*^1^*Significance set to p ≤ 0.05 values in bold denote significance.

95%CI, 95% Confidence Interval; VIF, Variance Inflation Factor; W, Watts; s, seconds; R², Coefficient of Determination; Statistic, F-Statistic; Cohen's f², Effect Size of Regression Model; 7SPP, 7-Stroke Peak Power; W_7SPP_, 7-Stroke Peak Power output (W); W_20_, 20-Second mean power output (W); W_60_, 60-Second mean power output (W); Exp, On-Water Rowing Experience (years).

The athlete with the fastest T_2000_ demonstrated a large leverage value when reviewing the Cook’s distance plot for each model and identified as a potential influential outlier (Aguinis et al., 2013; Zanin et al., 2024). Influential outliers are defined as potentially accurate data points which lie at a distance from other data points and can affect the conclusions of the study (Aguinis et al., 2013; Zanin et al., 2024). Each linear model was re-run without this athlete to determine if this outlier drastically influenced the results of each model. After removal, the R^2^, VIF, and f^2^ values were reduced for every model indicating how responsive the models were to a change in W_7SPP_, W_20_, and W_60_. Per recommended best practice (Aguinis et al., 2013; Zanin et al., 2024), all of the models were reproduced with the outlier removed; thus, helping to provide greater context and proper inference when evaluating this sample of female rowers against a larger rowing population. **Table 6** provides a summary of all models with the influential outlier removed.

**Table 6:** Summary and comparison of all linear regression models with influential outlier removed.

	7SPP			20-s			60-s			20-s + 60-s 2k time				20-s + 60-s 2k velocity				7SPP + 20-s + 60-s 2k time				7SPP + 20-s + 60-s 2k velocity			
Characteristic	Beta	95% CI	p-value*^1^*	Beta	95% CI	p-value*^1^*	Beta	95% CI	p-value*^1^*	Beta	95% CI	p-value*^1^*	VIF	Beta	95% CI	p-value*^1^*	VIF	Beta	95% CI	p-value*^1^*	VIF	Beta	95% CI	p-value*^1^*	VIF
(Intercept)	533.3569	499.6785, 567.0353	**<0.001**	551.4534	518.5397, 584.3672	**<0.001**	570.6805	537.2943, 604.0667	**<0.001**	570.8406	537.0040, 604.6772	**<0.001**		3.7235	3.3714, 4.0755	**<0.001**		574.3989	539.4163, 609.3814	**<0.001**		3.7347	3.3672, 4.1022	**<0.001**	
W7SPP	-0.1437	-0.2122, -0.0751	**<0.001**															-0.0424	-0.1413, 0.0565	0.4	2.92	-0.0001	-0.0012, 0.0009	0.8	2.92
W20				-0.2333	-0.3199, -0.1468	**<0.001**				-0.0294	-0.2033, 0.1445	0.7	4.70	-0.0006	-0.0024, 0.0012	0.5	4.70	0.0274	-0.1918, 0.2465	0.8	7.40	-0.0004	-0.0027, 0.0019	0.7	7.40
W60							-0.3116	-0.4079, -0.2152	**<0.001**	-0.2798	-0.4915, -0.0681	**0.011**	4.70	0.0029	0.0007, 0.0051	**0.012**	4.70	-0.2924	-0.5070, -0.0778	**0.009**	4.79	0.0028	0.0006, 0.0051	**0.015**	4.79
R²	0.328			0.446			0.537			0.539				0.357				0.548				0.358			
Adjusted R²	0.310			0.431			0.525			0.513				0.321				0.510				0.303			
Statistic	18.0			29.8			42.9			21.0				9.99				14.2				6.51			
Cohen's f²	0.4874			0.8064			1.1601			1.1671				0.5552				1.2141				0.5582			

*^1^*Significance set to p ≤ 0.05 values in bold denote significance.

95°/oCI, 95°/o Confidence Interval; VIF, Variance Inflation Factor; W, Watts; s, seconds; R^2^, Coefficient of Determination; Statistic, F-Statistic; Cohen's f^2^, Effect Size of Regression Model; 7SPP, 7-Stroke Peak Power; W_7spp_, 7-Stroke Peak Power output (W); W_20_, 20-Second mean power output (W); W_60,_ 60-Second mean power output (W).

The 7SPP had the highest ICC^3,1^ values (0.854, 95%CI = 0.778 to 0.912) indicating good to excellent reliability but it also had the highest TE (27.8 W, 95%CI = 23.6 to 33.9 W) and MDC_95_ (77.1 W, 95%CI = 65.4 – 93.9 W) and second highest CV% (6.43%, 95%CI = 5.43 – 7.88%). In other words, a change of 77.1 W would be required to be 95% confident a true change occurred. Conversely, the 60-s test had the worst ICC^3,1^ values (0.776, 95%CI = 0.671 to 0.861) indicating moderate to good reliability. However, it had the lowest TE (19.6 W, 95%CI = 16.6 to 23.9 W), MDC_95_ (54.5 W, 95%CI = 46.1 to 66.2 W) and CV% (6.04%, 95%CI = 5.10 to 7.40%). **Table 1** provides further details for the Test-Retest Reliability of the 7SPP, 20-s, and 60-s tests across all four test dates.

To evaluate reliability between sessions a pairwise comparison was conducted; please see **Table 2**. Surprisingly, the 60-s test showed the lowest ICC^3,1^ value for Date 2 vs. Date 4 (ICC = 0.592, [0.256, 0.800]) while the 7SPP test showed the highest ICC^3,1^ value for Date 3 vs Date 4 (ICC = 0.946, [0.892, 0.973]). Importantly, the TE and MDC_95_ for 7SPP showed the biggest range (TE = 16.2 – 37.8, MDC_95_ = 44.9 – 104.8) which again indicates an athlete has to have a substantial change in test score to be confident there is a clear change that occurred.

Analysis of the 7SPP within-session reliability is presented in **Table 7**. Across all four test dates mean CV% ranged 2.07 - 4.45%, indicating strong reliability for this test when evaluating consistency at the individual athlete level (Turner et al., 2015). While the majority of individual athlete-session CV% for all test dates was < 5%, a small number of trials exceeded this threshold. So, while **Tables 1** and **2** evaluates the reliability across and between all four test dates **Table 7** allows us to look at within athlete reliability which shows the 7SPP test is quite reliable.

**Table 7:** 7-Stroke peak power within-session trial reliability.

Test Dates				
Athlete ID	2023-08-29	2023-09-27	2023-11-30	2024-01-23
WROW001	3.75	5.29	2.48	5.93
WROW002	4.04	2.27	1.08	2.66
WROW003	4.99	0.44	1.95	1.91
WROW004	3.19	—	2.02	4.70
WROW005	4.71	1.44	1.21	1.95
WROW006	4.45	3.16	1.56	1.37
WROW007	2.56	—	1.87	2.53
WROW008	1.44	—	0.97	1.47
WROW009	2.56	14.56	0.84	1.53
WROW010	0.25	—	—	3.40
WROW011	2.70	5.34	0.42	3.98
WROW012	—	2.89	1.71	2.00
WROW013	2.63	2.08	3.52	3.31
WROW014	0.44	2.59	—	3.01
WROW015	7.48	—	2.53	0.62
WROW016	0.38	1.63	0.70	1.36
WROW017	—	6.64	1.22	3.30
WROW018	2.46	2.87	0.84	3.18
WROW019	—	2.50	3.92	1.47
WROW020	3.09	—	1.45	1.63
WROW021	—	14.69	—	2.31
WROW022	2.57	—	2.34	1.25
WROW023	—	1.82	0.99	3.10
WROW024	2.98	—	1.92	2.52
WROW025	3.46	—	2.92	3.96
WROW026	—	16.24	2.90	3.55
WROW027	1.53	1.91	—	4.64
WROW028	—	1.98	2.05	2.86
WROW029	2.94	—	2.18	3.47
WROW030	—	4.73	—	5.30
WROW031	5.24	—	0.96	0.54
WROW032	—	4.42	1.84	6.98
WROW033	2.61	2.94	—	4.36
WROW034	3.03	—	3.99	3.59
WROW035	2.34	—	1.86	3.46
WROW036	1.40	—	4.52	0.17
WROW037	—	2.42	6.66	17.96
WROW038	1.12	—	0.76	2.62
WROW039	—	1.93	—	3.20
WROW040	1.79	—	—	3.31
N	29	24	32	40
Mean CV%	2.83	4.45	2.07	3.26

– denotes athlete was absent or did not complete all three trials on that date.

CV(%) = (SD / Mean) x 100, where SD and Mean are computed across an athlete's three trial scores within each session.

Mean CV% reflects the average within-athlete trial consistency across 3 trials per session. It does not represent between-athlete variability. See **Table 2** for between-athlete heterogeneity.

Coefficient of Variation (%) Across 3 Trials per Test Date, by Athlete.

**Figure 3:**
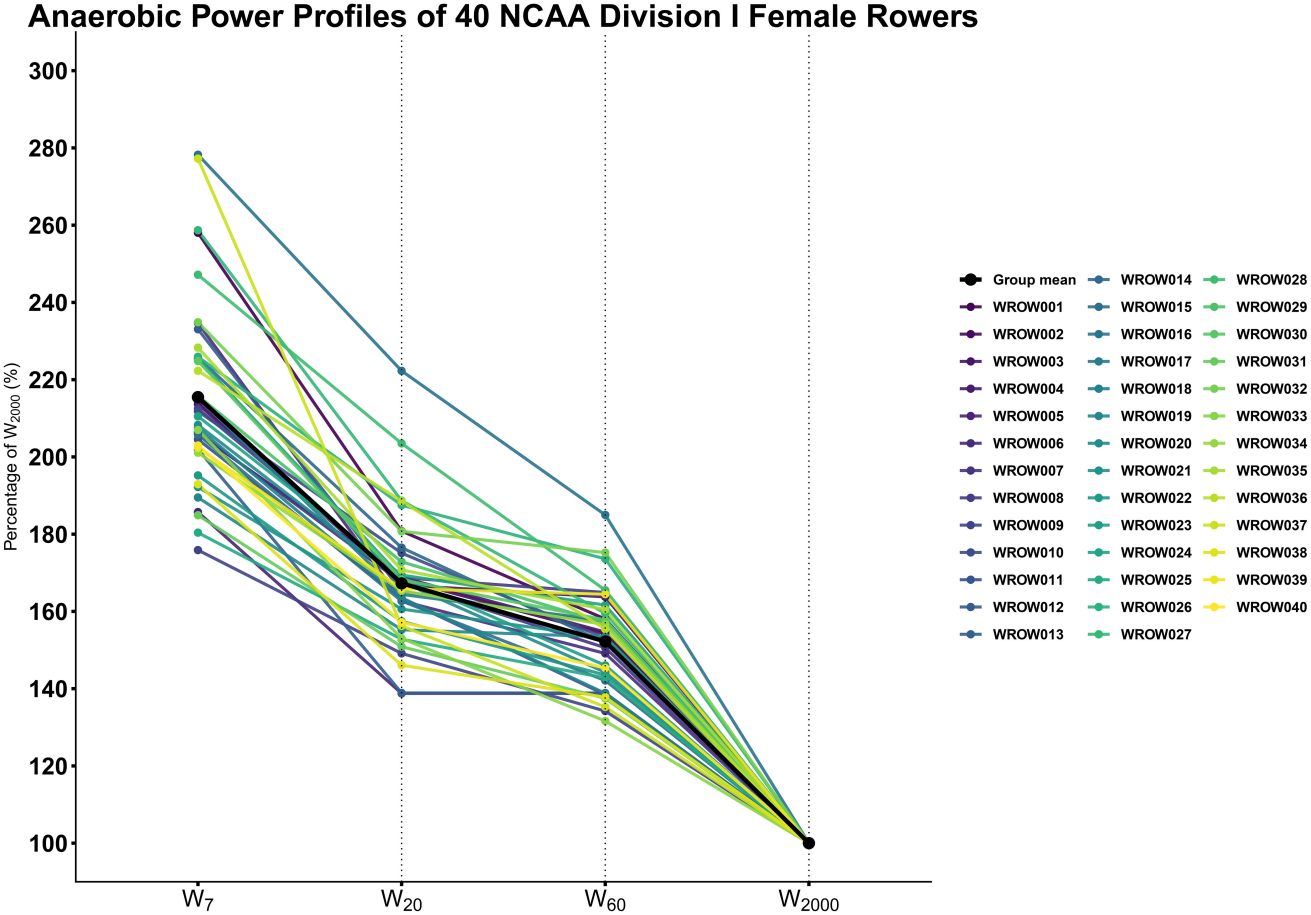
Anaerobic power profiles of 40 NCAA DI female rowers.

Finally, **Figure 3** shows the anaerobic profile of all 40 female rowers with W_7SPP_, W_20_, and W_60_ represented as a percentage of W_2000_ (2,000-meter watts = 100%).

## Discussion

The purpose of this study was to determine if W_7SPP_, W_20_, and W_60_ can serve as a predictor of 2kerg performance in a cohort of female DI rowers and apply the stepwise multiple regression models by Cerasola et al. (2022). Secondary aims were to determine if rowing experience improves the strength of any prediction models and to explore the intra-day and inter-day reliability of these tests.

The main practical finding of this study was that simple single linear regression models for W_60_ were the best single predictor for 2kerg performance; this is further supported by the high multicollinearity (VIF > 5) present in the multiple linear regression models. Additionally, stepwise multiple regression models that included W_20_ and W_60_ in addition to W_7SPP_ did not result in a better predictive model. To the best of our knowledge, this is only the second study to include a rowing cohort with a wide range of experience building off prior work by Legge and colleagues (Legge et al., 2024b). Our cohort’s on-water rowing experience ranged from 0–9 years, including athletes’ who were walking on to the WRow Team (had not rowed prior to college) to athletes with international rowing experience. Our cohort starkly contrasts the homogeneous cohorts that Cerasola (Cerasola et al., 2020; Cerasola et al., 2022), Ingham (Ingham et al., 2002), Astridge (Astridge et al., 2025), and Jensen (Jensen, 2007; Rowing W, 2019) investigated. The wide range of experience in our cohort could explain some of the reductions in explained variance compared to the prior investigations which focused exclusively on elite rowers at the junior and international competitive levels. We believe physiological capacity is a major determinant in why our model variance (R^2^) is lower compared to the elite rowing cohorts. In simple terms, our cohort had an average 2kerg time of 7:42.3 ± 17.5 (range: 6:58.8 to 8:25.7). In comparison, Astridge’s female rowers had an average 2kerg of 7:03.3 ± 9.7 seconds - about 5 seconds slower compared to the best athlete's score from our study. Cerasola’s cohort was an average of 15.8 years and average 2kerg was 6:58.5 ± 23.1 seconds (Cerasola et al., 2022) which is again impressive physiological capacity at that age.

The regression model of Cerasola, V_2000_ = 2.795 – (0.0005303 * W_20_) + (0.004680 * W_60_) resulted in a predicted speed of 5.67 ± 0.1 m/s and actual achieved speed of 5.49 ± 0.1 m/s. Our cohort’s predicted speed was 4.34 ± 0.1 m/s using the equation (V_2000_ = 3.244 – (0.0002892 * W_7PP_) – (0.0001972 * W_20_) + (0.0029436 * W_60_) and their actual speed was 4.3 ± 0.2 m/s, which are very similar to one another. This may be potentially attributed to the larger sample size we had in our study. Regardless, our findings illustrate the strong relationship and predictive capability that these short anaerobic tests have on 2kerg performance which aligns with the previous work of Cerasola (Cerasola et al., 2020; Cerasola et al., 2022), Cataldo (Cataldo et al., 2015), Ingham (Ingham et al., 2002), Astridge (Astridge et al., 2025), Šmída (Šmída et al., 2017), and Jensen (Jensen, 2005; Jensen, 2007; Rowing W, 2019). However, it also may indicate these tests are highly sensitive to elite rowers (high physiological capacity) and potentially not as accurate for athletes with less developed physiology. This has important testing and monitoring implications for rowing coaches and sport scientists.

First, previous work has shown the importance and value of these tests in youth rowers, and that they may be used to identify athletes who may have long-term success in rowing as well as to monitor positive adaptations to the training program (Legge et al., 2024b; Cerasola et al., 2022; Cataldo et al., 2015; Cerasola et al., 2020; Mikulić et al., 2009). Second, a major strength of these tests is that they are non-invasive, take less time to implement, and are easily scalable to large teams in comparison to maximal oxygen consumption or blood lactate tests (Cosgrove et al., 1999; Riechman et al., 2002). This provides more opportunities to implement these tests with greater frequency across an annual cycle.

Our cohort showed a large positive relationship between W_7SPP_ and W_2000_ (r = 0.66, p < 0.001), consistent with Ingham et al.'s findings with elite female rowers (r = 0.74, p < 0.001). While the association between W_7SPP_ and W_2000_ was lower in our cohort, this likely reflects the broader range of experience in our study; athletes in our cohort represent a population of DI rowers with a wide range of experiences and physiological capacities in comparison to elite females who made the ‘A’ final at the World Rowing Championships (Ingham et al., 2002). Additionally, it is expected that the elite rowers will have more rowing experience and our results demonstrated that this variable boosted the explained variance (R^2^) for all models. The female rowers in our study produced 492.0 ± 70.6 W for the 7SPP test whereas the junior and elite female rowers in Legge et al.’s study produced 403 ± 43 W and 525 ± 64 W, respectively. Furthermore, Legge utilized the McKay classification framework to distinguish the participants, ranking the elite rowers as world class (Tier 5) and the junior rowers as a combination of developmental and national level (Tiers 2 and 3) (McKay et al., 2022; Legge et al., 2024b). Taken together, our cohort’s ranking and physiological capacity is closer to that of the female elites, as they fall within 1 standard deviation of their W_7SPP_ which speaks to the quality of the participants in this investigation.

### Reliability

Several studies have investigated the reliability of these short anaerobic capacity tests. Most recently, Astridge and colleagues evaluated the ICC, standard errors of measurement (SEM), and TE. When comparing the between session reliability for the first trial of the MPP test on three separate days (24 hours apart) the mean ICC was 0.93 for males (range 0.92 – 0.95) and 0.98 for females (range 0.97 - 0.99) showing near perfect ICC values (Astridge et al., 2025). The CV% between sessions was also incredibly consistent for males (mean = 3.2%, range = 3.0 – 3.5%) and females (mean 2.4%, range = 2.3 – 2.5%) and SEM was low for both males (mean = 17.7 W, range = 16 – 19.4 W) and females (mean = 7.1 W, range = 5.6 – 9.8 W). Thus, Astridge et al.’s findings indicate that this cohort of athletes was incredibly consistent day-to-day when performing the MPP test. Nugent et al. used a load cell to evaluate the intra-session and inter-session reliability of the 7SPP test in 10 male national level Irish rowers (Nugent et al., 2019). Peak Power (W) intra-session reliability showed excellent agreement with intraclass correlation coefficient (ICC) of 0.99 (95%CI = 0.97-1.0), CV% of 1.1% (95%CI = 0.8 – 1.8%), and SEM of 8.5 W (Nugent et al., 2019). Inter-session reliability for Peak Power again showed excellent agreement with an ICC of 0.96 (95%CI = 0.88 - 0.99), CV% of 2.2% (95%CI = 1.6 – 3.4%), and SEM of 15.4 W (Nugent et al., 2019). In comparison, our cohort’s inter-session ICC values were 0.854 (95%CI =0.778 – 0.912), CV% of 8.01% (95%CI = 6.80 – 9.75%), and TE of 27.8 W (95%CI = 23.6 – 33.9 W). However, when evaluating within-session CV% for the 7SPP test at an individual level, the trial range was between 0.17 – 17.96% for all tests, but mean CV% range from 2.07 – 4.45%. A higher CV% indicates greater variability between repeated measures and less consistency across repeated measurements (Shechtman, 2013). The varied levels of physiology, experience, and training/competition level may be factors that contribute to the differences in reliability statistics in our cohort compared to prior studies. For coaches, this may make it difficult to interpret results and determine if there is a meaningful change in performance after a training block. To our knowledge, there is no other direct comparisons available for the 20-s and 60-s within or between session reliability.

There is value in testing and monitoring all three anaerobic tests as all demonstrated a high coefficient of determination (R^2^) to 2kerg performance. Based upon our investigation the 60-s test was the strongest single correlate and predictor to 2kerg performance. Our study, as well as previous investigations, have focused almost exclusively on the physiological capacity of the rowers and have not considered the validity and reliability of the Concept2 RowErg. The Concept2 RowErg has been shown to underestimate power by approximately 25 W when compared to a strain gauge (Boyas et al., 2006). A recent investigation by Treff et al. used a motorized rig and an external power measurement system (load cell) to compare against the derived power from the Concept2 RowErg and to allow for more precise control over force output and stroke rate. They found that when evaluating multiple series of 50 strokes that the inclusion of the first 5 strokes (from a dead stop) resulted in a measurement error of 2.5-4.3% (Treff et al., 2022). However, when removing the first 5 strokes from analysis this measurement error was reduced to 0.2 – 1.9% (Treff et al., 2022). Furthermore, they found that the random error of measurement increased considerably, approximately 10%, during rowing series where stroke rate fluctuated greatly (Treff et al., 2022). This investigation was also recently repeated using the RowPerfect3 (RP3) which is another popular rowing ergometer often used in training. Power in the first seven strokes was underestimated 2-83% compared to the external reference system and 4.7 – 5.3% across all 50 strokes (Mentz et al., 2025). However, once the first 7 strokes were eliminated, the differences were reduced to 0.9 – 1.7% (Mentz et al., 2025).

This additional context highlights important practical considerations for coaches and sport scientists if they choose to use these anaerobic tests. First, we must reiterate that we tested the reliability of the test being performed by a rower not the reliability of the Concept2 RowErg. Previous research by Nugent et al. shows that when using an external load cell the 7SPP has excellent intra-and-inter session reliability (Nugent et al., 2019). However, since the 7SPP and 20-s tests are short, they are exceptionally prone to an underestimation of actual power due to the huge difference in the first 5 strokes of these tests. Additionally, rowers perform the 7SPP and 20-s tests with stroke rate uncapped, meaning there will be additional variability and error in the Concept2 PM5 monitor due to rowers performing across unsteady stroke rates (Treff et al., 2022; Mentz et al., 2025). Interestingly, some testing protocols for peak power have had athletes take building strokes prior to their maximal strokes (Ingham et al., 2002; Metikos et al., 2015; Astridge et al., 2025). Astridge highlights that lead in strokes (five in their study) improves the reliability of the test and allows the rower to consistently perform at 40 strokes per minute. While this is true, this approach may be a tradeoff, in that the test does not mimic the conditions of an on-water 2k race, which begins from a dead-stop in the starting blocks. Regardless, if coaches still use the 7SPP and 20-s they must take into account that there will be increased variability from both the athlete and testing ergometer. Our recommendation is to use the 60-s test as this will have less noise and provide more accurate data to coaches and sport scientists and mitigate the risk of underestimating the actual anaerobic power capacity of the athletes. If coaches wish to use the 7SPP and 20-s tests they should use an external load cell to measure the actual force generated and ensure precise measurements.

### Anaerobic profile

When all three anaerobic capacity tests were expressed as a percentage of W_2000_, W_7SPP_ showed the highest values (215.5% ± 23.9%) followed by W_20_ (167.3% ± 15.9%) and W_60_ (152.2% ± 12.1%). Our results are remarkably similar to those of Cerasola and colleagues who reported 168.1% ± 14.1% for W_20_ and 153.1% ± 11.3% for W_60_ (Cerasola et al., 2022). This also aligns with values reported by Jensen, that we mentioned earlier, 100-meters showed a mean power of 175% ± 17%, 174% ± 16%, and 188% ± 16% relative to W_2000_ for the Danish, Italian, and USA elite male rowers, respectively (Jensen, 2007). Additionally, for the 60-s test the mean power was 154% ± 12%, 150% ± 8%, and 149% ± 3% relative to W_2000_ for the Danish, Italian, and USA elite male rowers, respectively (Jensen, 2007). Taken together, these findings continue to provide evidence that rowers should be able to achieve an approximate percentage of their W_2000_ when performing these tests.

### Limitations

One major limitation pertains to our basis for comparison; our study is comparing female university athletes to studies conducted in junior and male national level athletes. Future research comparing our results to cohorts of females competing at the junior and senior elite levels would provide additional insights. Additionally, our cohort consists of athletes within a single university rowing program; it would be beneficial to conduct a similar study across a broader female cohort to see how the results align. In our analysis, the addition of multiple independent variables has the potential to increase multicollinearity. This was evident in the multiple regression models, where two independent variables demonstrated high-intercorrelation (i.e. W_20_ and W_60_, r = 0.91). Thus, we believe this further indicates the use of a single anaerobic capacity test to predict 2kerg performance. We acknowledge that the temporal gap between the final anaerobic test date and 2kerg could have potentially influenced the fitness of the athletes. However, we can verify from the training program files that the athletes were following a polarized TID structure prior to the January 23^rd^ anaerobic capacity test date. So, there was not a pronounced shift in the TID in the lead up to either test time point. Finally, we did not control for diet and/or ergogenic aids during this study. It is possible that caffeine could influence performance on some of these tests.

## Conclusion

The 60-Second maximal effort test displayed the greatest predictive capability of all anaerobic capacity tests. This time-efficient anaerobic capacity test can be used to monitor rowing performance and indicate potential improvements in 2,000-meter time trial performance as well as positive adaptations to the training program. Coaches and rowers should be cautious in implementing the 7-Stroke Peak Power and 20-Second tests due to the high error from the Concept2 RowErg across these short distances. An external load cell is recommended if testing at these distances to ensure accuracy.

## Data Availability

The raw data supporting the conclusions of this article will be made available by the authors upon reasonable request, without undue reservation.
